# Dynamic localisation of Ran GTPase during the cell cycle

**DOI:** 10.1186/1471-2121-10-66

**Published:** 2009-09-18

**Authors:** James RA Hutchins, William J Moore, Paul R Clarke

**Affiliations:** 1 Biomedical Research Institute, College of Medicine, Dentistry and Nursing, Ninewells Hospital and Medical School, University of Dundee, Dundee, DD1 9SY, UK; 2 Research Institute of Molecular Pathology (IMP), Dr. Bohr-Gasse 7, A-1030 Vienna, Austria; 3 Division of Gene Regulation and Expression, Wellcome Trust Biocentre, College of Life Sciences, University of Dundee, Dundee, DD1 5EH, UK

## Abstract

**Background:**

Ran GTPase has multiple functions during the cell division cycle, including nucleocytoplasmic transport, mitotic spindle assembly and nuclear envelope formation. The activity of Ran is determined by both its guanine nucleotide-bound state and its subcellular localization.

**Results:**

Here, we have characterised the localisation and mobility of Ran coupled to green fluorescent protein (GFP) during the cell cycle in live human cells. Ran-GFP is nuclear during interphase and is dispersed throughout the cell during mitosis. GFP-Ran^Q69L^, a mutant locked in the GTP-bound state, is less highly concentrated in the nucleus and associates with nuclear pore complexes within the nuclear envelope. During mitosis, GFP-Ran^Q69L ^is excluded from chromosomes and localizes to the spindle. By contrast, GFP-Ran^T24N^, a mutant with low affinity for nucleotides, interacts relatively stably with chromatin throughout the cell cycle and is highly concentrated on mitotic chromosomes.

**Conclusion:**

These results show that Ran interacts dynamically with chromatin, nuclear pore complexes and the mitotic spindle during the cell cycle. These interactions are dependent on the nucleotide-bound state of the protein. Our data indicate that Ran-GTP generated at chromatin is highly mobile and interacts dynamically with distal structures that are involved in nuclear transport and mitotic spindle assembly.

## Background

Ran is a member of the Ras superfamily of small GTPases and has multiple roles in coordinating essential nuclear and mitotic processes in eukaryotes [1]. Like other GTPases, Ran exists in GTP-bound or GDP-bound conformations that differ in their molecular interactions. Conversion between these states requires the interaction of accessory proteins. Ran itself is mainly soluble and is concentrated in the nucleus by an active import mechanism. Ran-GTP is generated in the nucleus by RCC1, a chromatin-associated guanine nucleotide exchange factor specific for Ran (RanGEF). The intrinsic GTPase activity of Ran is very low but is stimulated greatly by cytoplasmic RanGAP1 in conjunction with Ran-GTP-binding domains of RanBP1 or RanBP2, a large nucleoporin (Nup358) that is located at the cytoplasmic side of the nuclear pore complex (NPC). The spatial separation of these regulators is thought to establish a high concentration of Ran-GTP in the nucleus and a low concentration in the cytoplasm [2].

Ran plays a central role in the nucleocytoplasmic transport of many macromolecules during interphase [3,4]. The high concentration of Ran-GTP in the nucleus promotes the disassembly of nuclear import complexes between proteins carrying a leucine-rich nuclear export signal (NES) and the transport factor Crm1, while dissociating nuclear import complexes formed between importins and cargo proteins carrying a lysine-rich nuclear import signal (NLS) [5]. In mitosis, when the controlled barrier of the nuclear envelope is broken down in animal cells, generation of Ran-GTP by chromosomal RCC1 plays a role in directing formation of the mitotic spindle [6]. At the end of mitosis, Ran controls the reformation of the nuclear envelope (NE) around chromatin [7-9] and the assembly of nuclear pore complexes (NPCs) [9,10].

Since the activity of Ran is determined by both its guanine nucleotide-bound state and its localisation, it is of interest to determine the relationship between these factors. This differential concentration of Ran-GTP across the nuclear envelope (NE) has been visualised indirectly using FRET (fluorescence resonance energy transfer) probes derived from Ran-GTP-binding domains in both *Xenopus *egg extracts and live mammalian cells [11-13]. In M-phase *Xenopus *egg extracts, a gradient of Ran-GTP concentrations decreasing away from metaphase chromosomes arranged on the spindle is observed [11]. In mammalian cells in mitosis, a Ran-GTP gradient is also observed, although the gradient is less marked than in *Xenopus *egg extracts [13]. While these experiments provide a dramatic illustration of the distribution of Ran-GTP concentrations within a cell, the method is limited in resolution and may not identify Ran-GTP localised at subcellular structures.

In fixed cells, Ran has been localised to specific structures including the NE and the mitotic spindle by either indirect immunofluorescence or tagging Ran with naturally a fluorescent protein [14-16]. However, results from fixed and extracted cells are potentially prone to artifactual condensation onto such structures. Live-cell imaging of Ran labelled with a fluorophore and injected into cells indicated that Ran associates dynamically with M-phase chromatin in eggs and embryonic cells, and to a lesser extent with mitotic chromosomes in a mammalian cell line, although the nucleotide-dependency of this interaction was unclear [17].

Here, we have determined the localisation of Ran expressed as a fusion with green fluorescent protein (GFP) at high resolution in living, unfixed cells in interphase and during progression through mitosis. To investigate the role of the GTP-GDP cycle of Ran in determining its localisation, we have also localised two mutants of Ran expressed as GFP-fusions, Ran^Q69L^, which is defective in GTP hydrolysis and therefore is predominantly in the GTP-bound form [18,19], and Ran^T24N^, which has reduced affinity for nucleotides and forms a stable complex with RCC1 [19]. We show that Ran-GTP does not associate with chromosomes but forms stable interactions with NPCs in interphase and the spindle during mitosis. These results support a model in which soluble Ran-GTP generated at chromosomes diffuses within cells to specific intracellular structures where it interacts transiently to carry out its functions.

## Results

### Localisation of Ran and its mutants in live cells

In order to study the sub-cellular localisation and dynamics of Ran during the division cycle, wild-type (WT) and mutant Ran proteins were expressed as GFP-fusions in cultured human cells. To assess the localisation of these fluorescent proteins with respect to nuclear or mitotic chromosomes, co-transfections were performed with a plasmid expressing red fluorescent protein (RFP) fused to histone H2B (RFP-histone). The localisation of these fluorescent proteins was then observed during stages of the cell division cycle by fluorescence microscopy using a DeltaVision Spectris microscopy workstation. Data is shown for U2OS cells, but consistent results were also obtained using HeLa and HEK293 cells.

**Figure 1:**
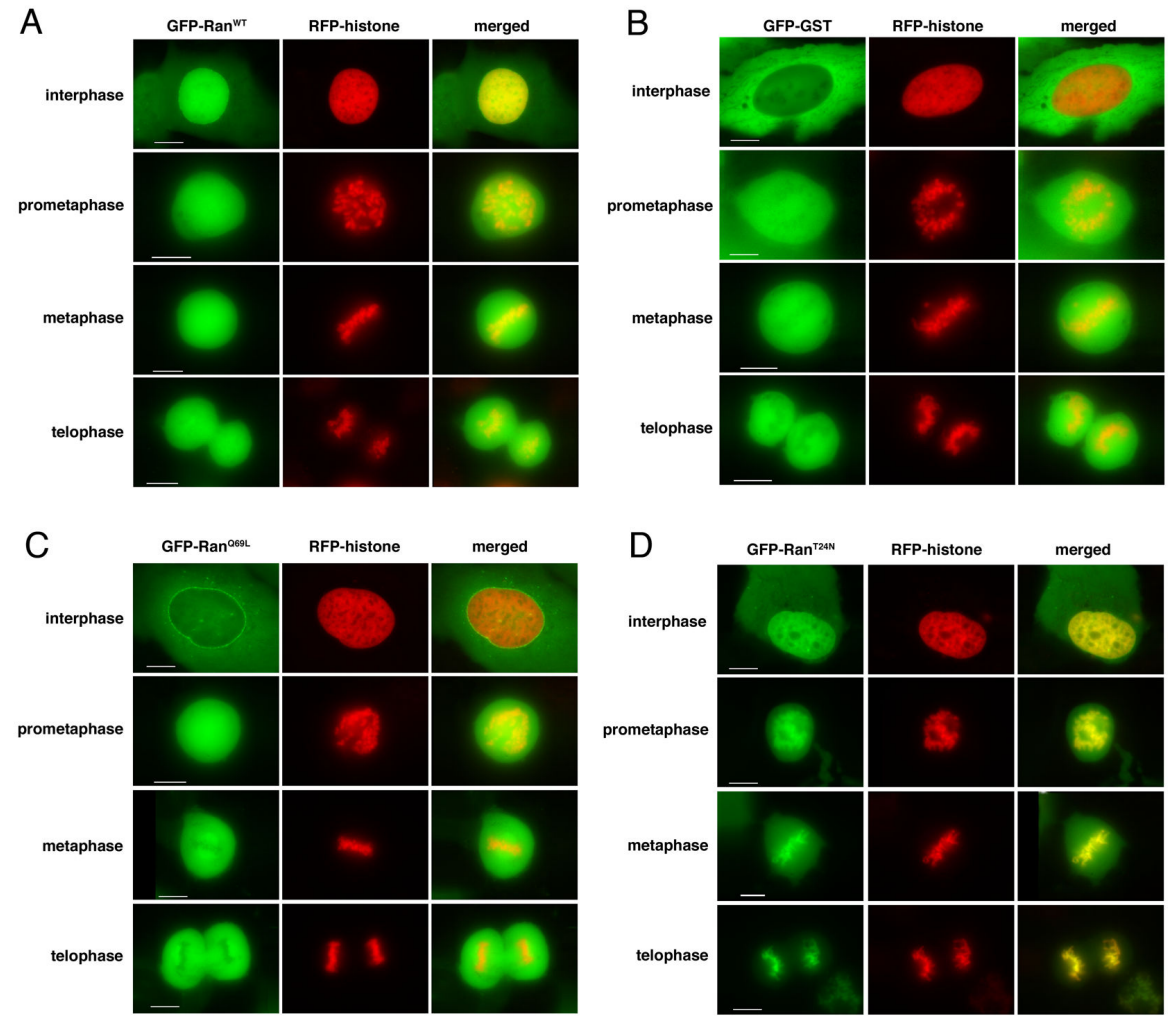
**Localisation of GFP-Ran proteins in live human cells**. Fluorescence images of live U2OS cells transfected with RFP-histone 2B and (A) GFP-Ran^WT^, (B) GFP-GST, (C) GFP-Ran^Q69L ^or (D) GFP-Ran^T24N ^showing GFP (green), RFP (red) and merged images. Representative images are shown for individual cells in interphase or during specific phases of mitosis that are readily identified by chromosome condensation state and organisation. Scale bars, 10 μm.

As expected, GFP-Ran^WT ^localised predominantly to the nuclei of live interphase cells, with low levels of cytoplasmic signal (Figure 1A). By contrast, a control fusion between GFP and glutathione-S-transferase (GFP-GST), which was predominantly cytoplasmic in all interphase cells (Figure 1B). The RFP-histone signal localised within nuclei with a dappled pattern typical of interphase chromatin, whereas nuclear GFP-Ran^WT ^displayed a diffuse localisation, suggesting that it is largely dispersed within the nucleoplasm. During mitosis, GFP-Ran^WT ^was widely and uniformly dispersed throughout cells (Figure 1A), whereas GFP-GST was clearly excluded from condensed chromosomes (Figure 1B). This suggests that, although GFP-Ran^WT ^is not concentrated on chromosomes, it does interact with mitotic chromatin.

The mutants of Ran, GFP-Ran^Q69L ^and GFP-Ran^T24N^, exhibited distinctly different localisations from GFP-Ran^WT ^and from each other. In interphase cells, the ratio of GFP-Ran^Q69L ^in the nucleus and the cytoplasm was variable between individual cells, with some cells showing a predominantly cytoplasmic localisation and others with a more pronounced nuclear localisation. However, there was a clear, punctate concentration of GFP-Ran^Q69L ^at the nuclear envelope in all interphase cells (Figure 1C). Mitotic cells expressing GFP-Ran^Q69L ^were less frequently observed in the culture, consistent with an inhibitory effect of this dominant mutant on cell cycle progression and entry into mitosis. However, those cells expressing GFP-Ran^Q69L ^and undergoing cell division showed that the protein was excluded from chromosomes and localised to the mitotic spindle from metaphase through to telophase (Figure 1C). GFP-Ran^T24N^, in contrast to the WT and Q69L proteins, showed a distinctly heterogenous nuclear localisation that exactly corresponded to RFP-histone, indicating an association with chromatin. GFP-Ran^T24N ^remained highly concentrated on chromatin throughout mitosis (Figure 1D).

### RanQ69L interacts with stably with NPCs during interphase

**Figure 2:**
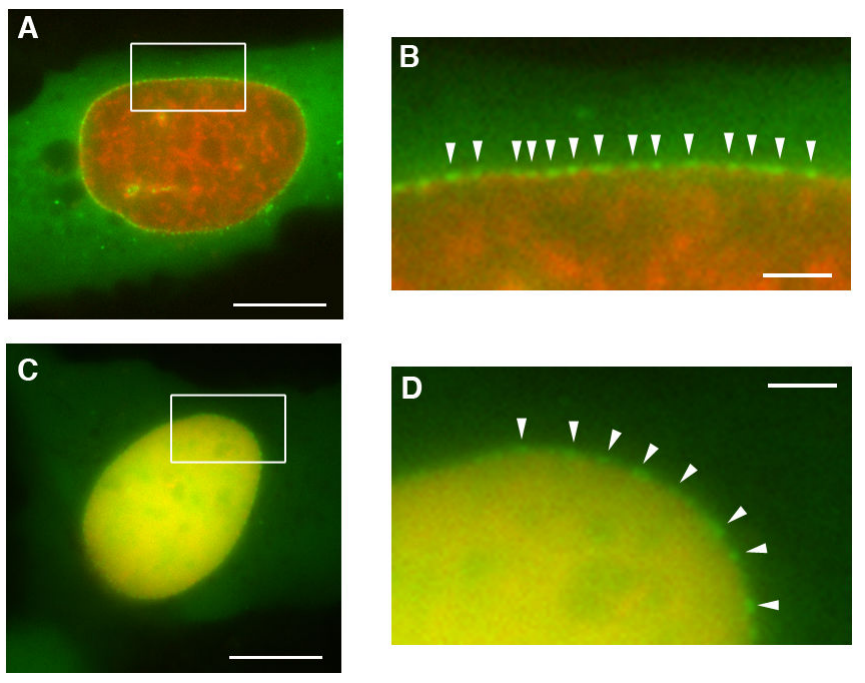
**Localisation of GFP-Ran^Q69L ^to nuclear pore complexes**. (A) Merged image of a live RFP-histone 2B/GFP-Ran^Q69L^-transfected U2OS cell exhibiting punctate nuclear rim staining. (B) 4× enlargement of the rectangular section highlighted in (A), showing the regular distribution of NE-associated foci that are most likely to be NPCs (white arrows). (C, D) Merged images of two live RFP-histone/GFP-Ran^WT^-transfected U2OS cells with multiple mini-nuclei. Scale bars, 10 μm (A,C) or 1 μM (B,C).

**Figure 3:**
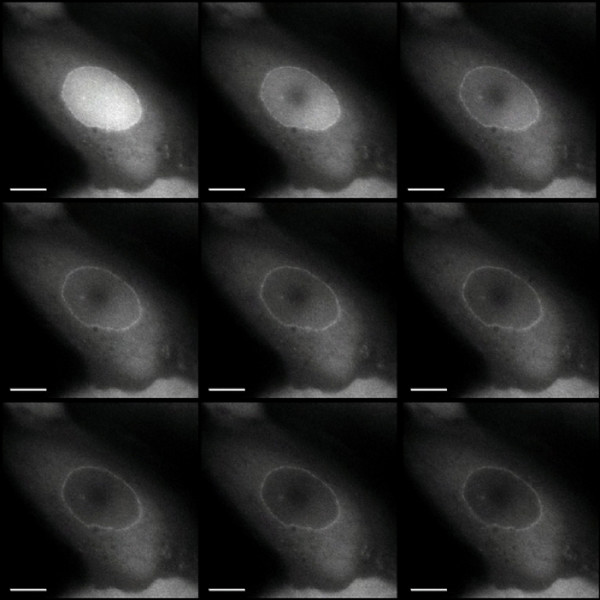
**FLIP analysis of GFP-Ran^Q69L ^**.Images of a cell expressing nuclear GFP-Ran^Q69L ^(top left) and after successive cycles of photobleaching within a defined spot within the nucleus (left to right, top to bottom). The cell was subjected to 100 cycles of laser-bleaching, each of 20 ms duration and images collected at 250 ms intervals are shown. See additional file 1: movie 1 for the original data used to perform this analysis.

Close examination indicates that the punctate localisation of GFP-Ran^Q69L ^at the NE is very likely to correspond to individual NPCs (Figure 2A, B). While this localisation was most apparent for GFP-Ran^Q69L^, it was also observed more weakly for GFP-Ran^WT ^(Figure 2C, D) and also for GFP-Ran^T24N ^(Figure 1D, data not shown). In a cell showing nuclear localisation of GFP-Ran^Q69L^, repetitive bleaching of a spot within the nucleus by a laser (fluorescence loss in photobleaching, FLIP) showed that the fluorescence signal was rapidly lost throughout the nucleoplasm, while the punctate NE signal persisted (Figure 3). This indicates that GFP-Ran^Q69L ^is highly mobile within the nucleus but relatively stably associated with NPCs.

### Relocalisation of RanQ69L during mitosis

**Figure 4:**
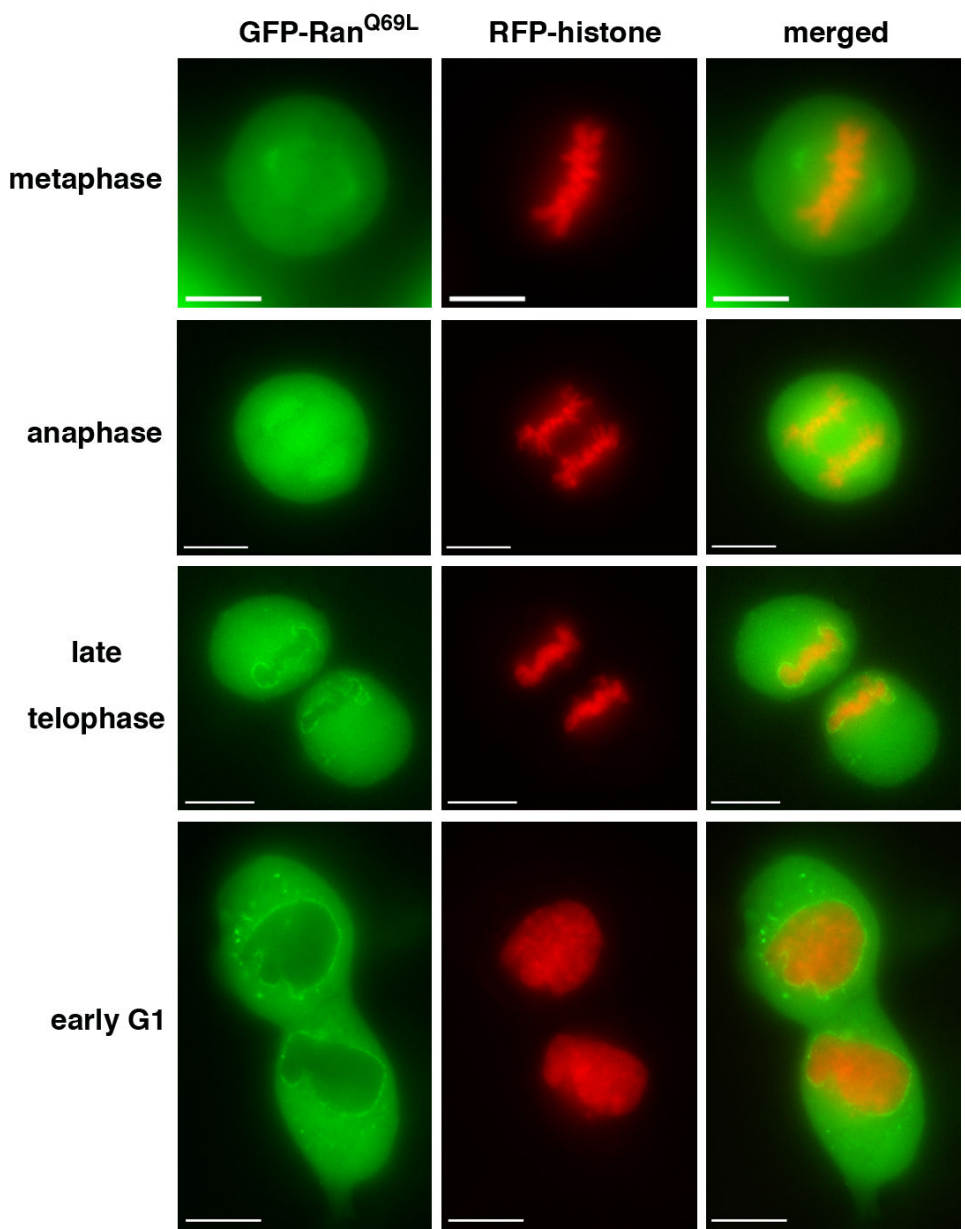
**Relocalization of GFP-Ran^Q69L ^during mitosis**. Fluorescence images of live U2OS cells transfected with RFP-histone 2B and GFP-Ran^Q69L ^showing GFP (green), RFP (red) and merged images. Representative images are shown for individual cells during specific phases of mitosis and early G1 that are readily identified by chromosome condensation state and organisation. Scale bars, 10 μm.

**Figure 5:**
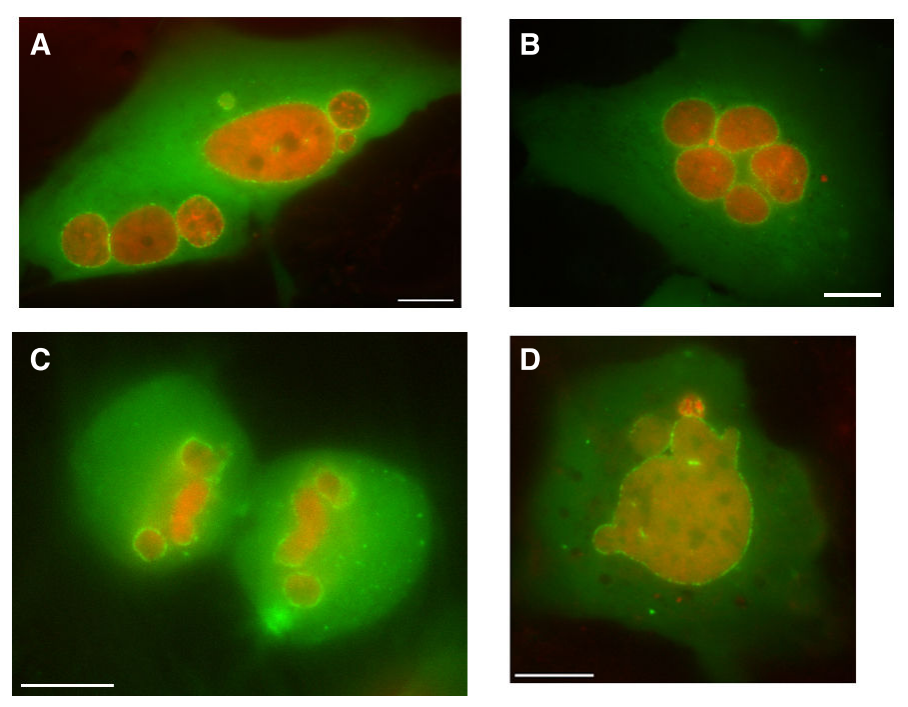
**Aberrant nuclear structures in cells expressing GFP-Ran^Q69L^**. Merged images of a live RFP-histone 2B/GFP-Ran^Q69L^-transfected U2OS cells exhibiting multiple nuclei (A,B), formation of NE around separate chromosome masses in telophase (C) and a lobed nucleus (D). Scale bars, 10 μm.

During progression through mitosis, the localisation of GFP-Ran^Q69L ^changed from the mitotic spindle in metaphase-anaphase to the reforming NE in telophase when the spindle is disassembled (Figure 4). In metaphase, GFP-Ran^Q69L ^was present on the mitotic spindle, with some concentration towards the poles. At anaphase, the spindle localisation persisted and some concentration was observed between the segregated chromosomes. Towards the end of mitosis, GFP-Ran^Q69L ^became relocalised in a punctate pattern to the reforming NE in telophase (Figure 4). Interphase cells expressing GFP-Ran^Q69L ^frequently exhibited a multinucleate phenotype (Figure 5A, B). A cell in late telophase (Figure 5C) showed a punctuate GFP-Ran^Q69L ^localisation around multiple chromosomal masses, suggesting that the multinuclear phenotype might be generated through aberrant NE formation or a failure to coalesce chromosomes at this stage of the cell cycle. Cells expressing GFP-Ran^Q69L ^alternatively showed a lobed nuclear phenotype (Figure 5D) and/or had punctate GFP-Ran^Q69L ^localisation in the cytoplasm (Figure 2, 5C).

### RanT24N associates with chromatin throughout the cell cycle

**Figure 6:**
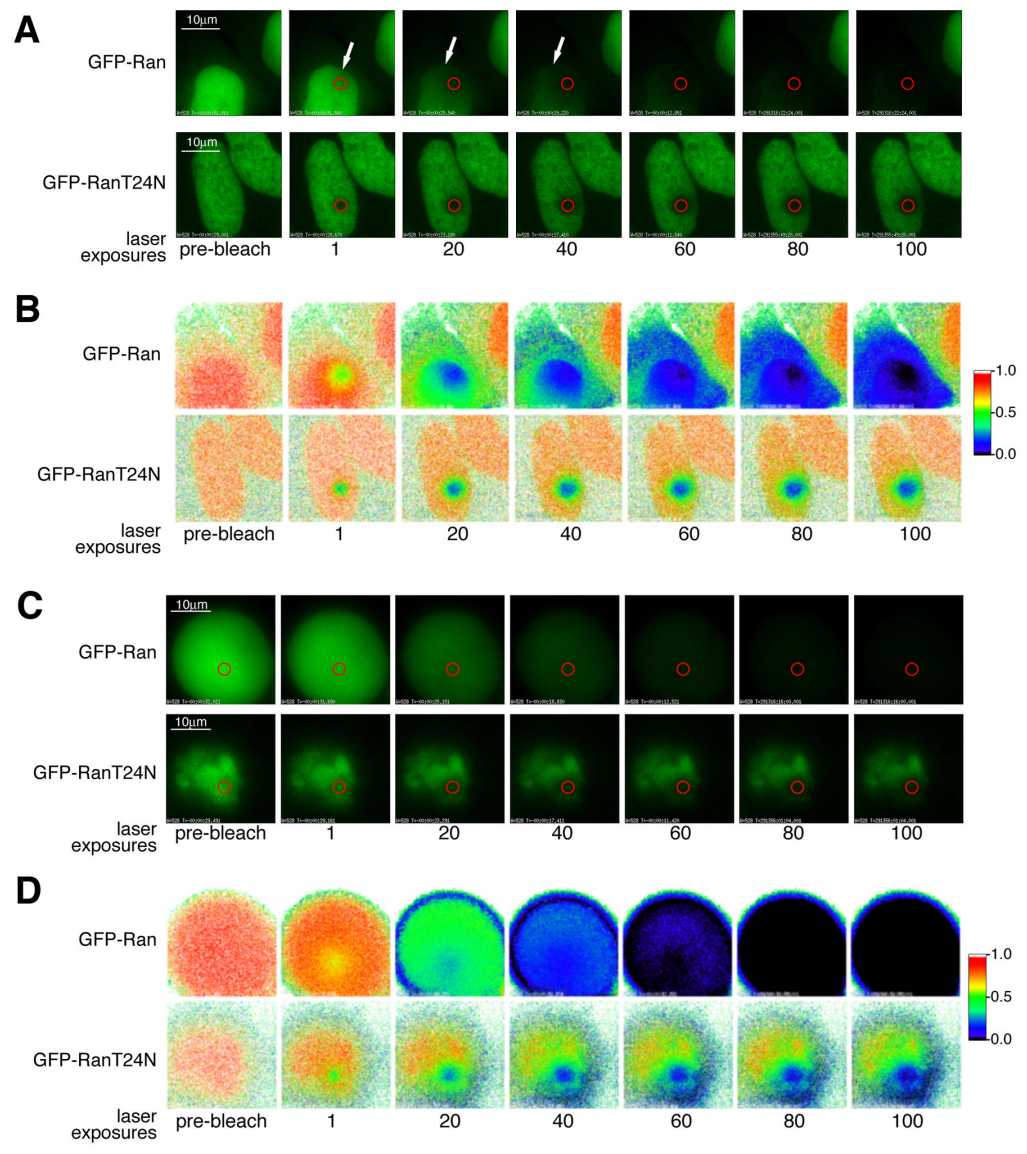
**FLIP analysis of GFP-Ran^WT ^and GFP-Ran^T24N^**. Successive images of individual cells expressing GFP-Ran^WT ^or GFP-Ran^T24N ^in interphase (A) or mitosis (C) after photobleaching within a defined spot within the nucleus for the number of exposures shown. (B,D) False-colour imaging showing the relative change in fluorescence for the cells shown in (A) and (C), from orange through green to blue for increasing change, illustrating bleaching of the fluorescence signal.

A relatively stable interaction between GFP-Ran^T24N ^and chromatin compared to GFP-Ran^WT ^was illustrated by FLIP experiments (Figure 6) in which a spot in the nucleus was repeatedly bleached and the loss of overall fluorescence signal was recorded (Figure 6A). Changes in fluorescence were converted to a false colour signal to illustrate the difference between proteins (Figure 6B). In the nuclei of cells expressing GFP-Ran^WT^, almost the entire nuclear signal was bleached after 100 pulses of the laser. There was also a slower reduction in the initially weaker fluorescence signal in the cytoplasm. These results show that GFP-Ran^WT ^is a highly mobile within the nucleus and it also exchanges with the cytoplasm. By contrast, GFP-Ran^T24N ^was only bleached in an area within the nucleus close to the bleach spot, showing that it is much less mobile (Figure 6A, B). Similarly, in mitotic cells in which GFP-Ran^WT ^is universally dispersed, repeated bleaching reduced the fluorescent signal almost uniformly across the cell (Figure 6C, D). This shows that GFP-Ran^WT ^is highly mobile in mitosis and interactions with structures such as chromosomes and the spindle are too transient to be observed by this method. On the other hand, bleaching of GFP-Ran^T24N^-expressing mitotic cells adjacent to the chromatin resulted in only a slow decline in the GFP-signal on chromosomes where it is mainly localised, indicating that GFP-Ran^T24N ^is stably associated with mitotic chromatin and only exchanges with the rest of the cell slowly.

### Dynamic mobility of Ran during the cell cycle measured by FRAP

**Figure 7:**
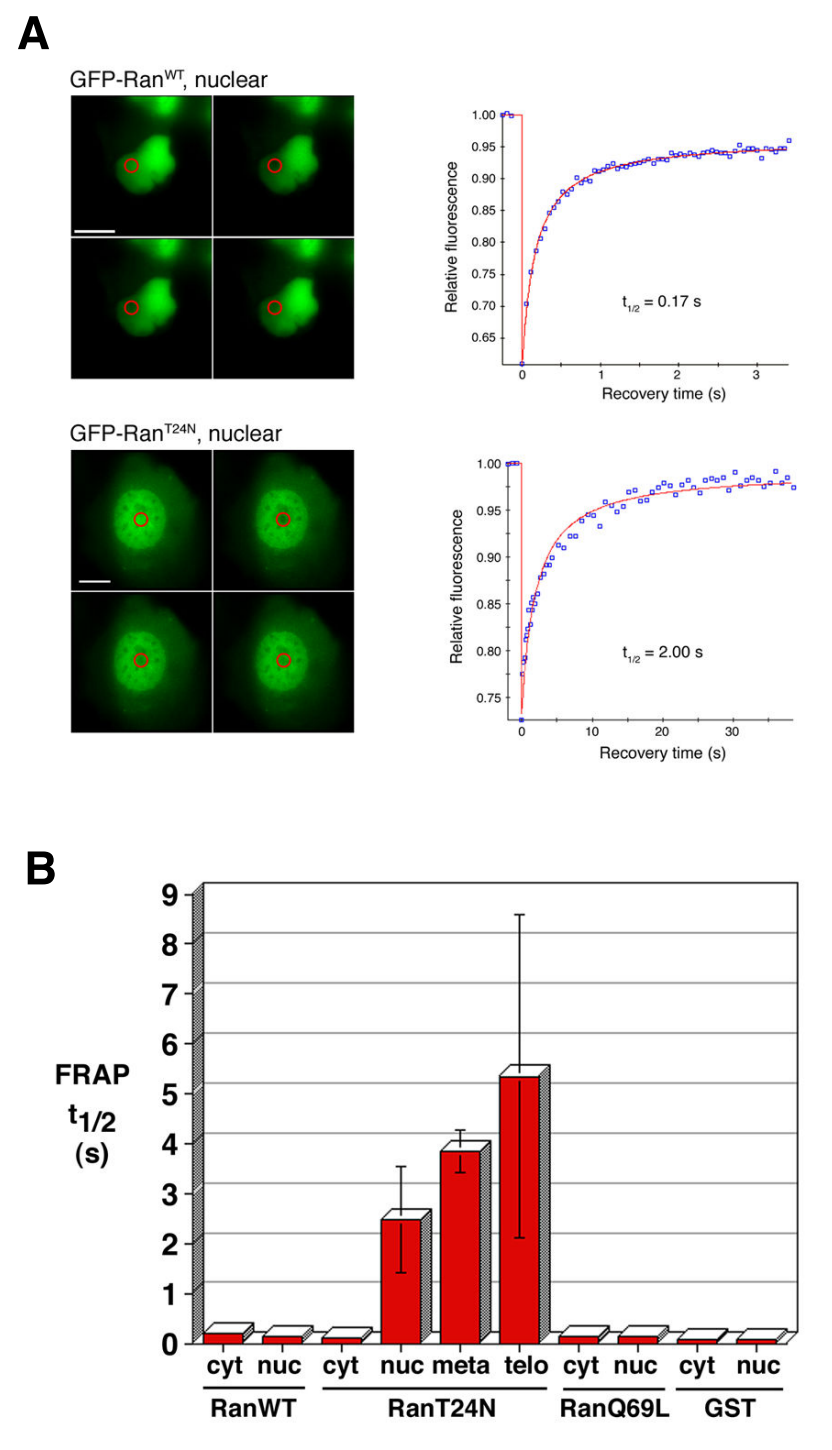
**FRAP analysis of GFP-Ran proteins**. (A) Example FRAP experiments focused on the nuclei of GFP-Ran^WT^- and GFP-Ran^T24N^-transfected U2OS cells. Left, fluorescence images of cells; in each panel, top left image shows a cell before bleaching then successive images left to right, top to bottom after bleaching. Scale bars, 10 μm. Right, plots showing recovery of the fluorescence signal in each case and the calculated t_1/2_. (B) Differences in FRAP t_1/2 _values between different forms of Ran, during progression through the cell cycle and in different subcellular compartments: cyt, interphase cytoplasm; nuc, interphase nucleus; meta, metaphase chromatin; telo, telophase chromatin.

To study the intracellular mobility of Ran quantitatively, in particular with respect to its interaction with chromatin in its GTP-bound and nucleotide-free forms, we performed fluorescence recovery after photobleaching (FRAP) experiments in GFP-Ran-transfected cells to determine the recovery half-times (t_1/2_) for the GFP-proteins (Figure 7). This value gives a measure of the mobility of the GFP-proteins within the compartment and can be used to infer the stability of interaction with structures, for instance chromatin. A typical experiment comparing GFP-Ran^WT ^and GFP-Ran^WT ^within the nuclei of interphase cells is shown in Figure 7A. The t_1/2 _value for Ran^WT ^of 0.17s indicates a high degree of mobility within the nucleus and therefore a weak interaction with chromatin. By contrast, t_1/2 _value for GFP-Ran^T24N ^was 2.00s, indicating a much slower mobility and therefore a more stable interaction with chromatin.

We carried out multiple such experiments on interphase cells expressing sufficient GFP-Ran in both the nucleus and the cytoplasm for photobleaching in both compartments (Figure 7B). Because GFP-Ran^WT ^and GFP-Ran^Q69L ^did not associate with chromosomes during mitosis, only GFP-Ran^T24N^-expressing cells were chosen for FRAP experiments on mitotic chromosomes. These FRAP studies showed both GFP-Ran^WT ^and GFP-Ran^Q69L ^to be extremely mobile in the interphase nucleus, with a mean t_1/2 _value (0.13 s) not significantly different from than that in the cytoplasm (0.19 s), and only marginally greater than that for GFP-GST in both compartments (0.07 s). By comparison, the FRAP t_1/2 _for RFP-histone H2B, a protein expected to be very stably associated with chromatin, was determined as being 50-60 s. In contrast, the mean FRAP t_1/2 _for nuclear GFP-Ran^T24N ^(~ 2.5 s) was nearly 20-fold greater than that for nuclear GFP-Ran^WT ^and GFP-Ran^Q69L^, and for cytoplasmic GFP-Ran^T24N^. During progression through mitosis the mobility of GFP-Ran^T24N ^decreased considerably, with the mean FRAP t_1/2 _rising to 3.85 s in metaphase, and 5.35 s in telophase.

## Discussion

The functions of Ran in different processes during the cell cycle are determined by both the guanine nucleotide-bound state and the localisation of the protein. Two divergent models have been hypothesised for the interplay of these conditions, particularly with regard to mitosis in animal cells when the controlled boundary between the chromosomes and cytoplasm is broken down [1]: one model is that Ran is soluble and the spatial organisation of its regulators associated with intracellular structures generates a gradient of Ran-GTP that provides spatial information [6]; a different model is that Ran stably interacts with structures within the cell to control localised reactions depending on its guanine nucleotide-bound state [20].

Here, we have used live-cell imaging techniques to determine the localisation of Ran in somatic human cultured cells. We find that wild-type Ran, which can freely convert between GDP and GTP-bound states depending upon its interaction with localised regulators, when expressed as a fusion with GFP is dispersed in the nucleus during interphase and also localises to NPCs. In mitosis, GFP-Ran is dispersed throughout the cell and no localisation to specific structures could be discerned, even using FLIP techniques to bleach mobile GFP-Ran molecules. Nevertheless, GFP-Ran was not excluded from chromosomes during mitosis, suggesting that it does interact transiently with chromatin.

To investigate the relationship between localisation and the nucleotide-bound state of Ran, we also used GFP-Ran stabilised in different nucleotide-bound states by mutation. A mutant that is locked in the GTP-bound state, Q69L, was less concentrated in the nucleus and showed prominent localisation to NPCs within the NE during interphase. The lack of concentration of the mutant into the nucleus is consistent with its inability to interact with the import factor NTF-2 even when it has GDP bound [21]. GFP-Ran^Q69L ^may also be sequestered by stable binding to components of the NPC, such as RanBP2 via its Ran-GTP binding domains. Ran^Q69L ^alters the conformation of NPCs formed in vitro in *Xenopus *egg extracts [22], suggesting that Ran-GTP binding to NPC components may modulate the structure and perhaps function of the NPC in nucleocytoplasmic transport. The similar localisation of GFP-Ran^WT ^to NPCs, albeit less pronounced, indicates that this is a normal if transient interaction for Ran. However, we found that GFP-Ran^T24N ^is also associated with NPCs in live cells, consistent with previous immunofluoresence of this mutant in fixed cells (Lounsbury, 1996), indicating that Ran can interact with NPCs independently of its GTP-bound state. This would be consistent with the ability of Ran to also interact with the NPC when in the GDP-bound conformation [23].

The lack of association between Ran^Q69L ^and chromatin means that it is unlikely that Ran-GTP forms stable interactions with chromatin-bound proteins either during interphase or in mitosis. However, we show that Ran-GTP in the form of GFP-Ran^Q69L ^localises to the mitotic spindle where it may interact with Ran-GTP-binding proteins. Concentration of GFP-Ran^Q69L ^on the spindle strongly suggests that Ran-GTP normally interacts transiently with this structure and may be released with GTP hydrolysis induced by RanGAP1.

The T24N mutant of Ran does not simply correspond to a GDP-bound conformation, since it is defective in binding either GTP or GDP [19], but it provides a useful comparison with Ran^Q69L ^since it is does not form interactions dependent on the GTP-bound conformation of Ran. Ran^T24N ^probably exists in cells mainly in a nucleotide-free form that makes a stable complex with the chromatin-associated guanine nucleotide exchange factor for Ran, RCC1. Ran^T24N ^stabilises the interaction of RCC1 with chromatin [24], probably through a conformational change that exposes the N-terminal tail of RCC1 [25]. Indeed, the localisation of GFP-Ran^T24N ^is very similar to that of RCC1 throughout the cell cycle [15]. We find that GFP-Ran^T24N ^associates with chromatin much more stably than GFP-Ran^WT^, although it is worth noting that its interaction is still dynamic, has kinetics similar those of RCC1 fused to GFP and is similarly stabilised during progression through mitosis from metaphase to telophase [26]. The localisation of GFP-Ran^T24N ^therefore is likely to represent the sites of generation of Ran-GTP by RCC1.

Unfortunately, no mutation has been identified that would stabilise Ran specifically in the GDP-bound conformation and so the localisation of Ran-GDP cannot yet be determined directly by this approach. The difference in chromatin interaction of GFP-Ran^WT ^and GFP-Ran^Q69L ^(only the latter is excluded from mitotic chromatin) may indicate that Ran-GDP interacts with chromatin in cells, as has been found in vitro in Xenopus egg extracts [9]. Alternatively, it may represent a transient nucleotide-free interaction with RCC1, although it is not expected that this interaction would be disrupted by the Q69L mutation [19], suggesting that Ran-GTP is rather actively excluded from mitotic chromatin.

Our results are consistent with the interplay between the nucleotide-bound state of Ran and its dynamic localisation, whereby soluble Ran-GTP is generated at chromatin by RCC1 and then rapidly diffuses throughout the compartment until it interacts with specific structures where it carries out localised functions. During interphase, Ran is concentrated in the nucleus by active import where it is predominantly GTP-bound, but it does interact with NPCs. During mitosis, Ran is dispersed throughout cells, but interacts with chromatin and the spindle. The interaction of Ran with structures within the cell may allow localised nucleotide cycling linked to the assembly/disassembly of immobilised protein complexes and control their function specifically at that site [20]. Such a mechanism might account for the action of Ran at kinetochores where RanBP2 and RanGAP1 are localised during mitosis [27,28].

Localisation of Ran to structures such as chromatin may alternatively be thought of as a mechanism to simply increase its concentration above a certain threshold, which when exceeded triggers complex formation, ensuring spatial organisation of the functions of Ran. Such a mechanism has been proposed for the function of Ran in nuclear envelope assembly in *Xenopus *egg extracts. In this system, chromatin recruits Ran, initially in its GDP-bound state [9]. The recruitment of Ran occurs prior to that of RCC1 [9] and can be independent of RCC1 [29]. The sufficiency of increasing the concentration of Ran to induce NE formation was demonstrated by the ability of Ran-coated beads to form NE around them in the complete absence of chromatin [7].

In a general model for the role of Ran-GTP in intracellular spatial organisation, soluble Ran-GTP concentrations provide an indicator of the presence of chromosomes, with a homogenous Ran-GTP concentration throughout the nucleoplasm during interphase. In mitosis, the high concentration of Ran-GTP in the vicinity of chromosomes relative to the rest of the cell provides a low-resolution spatial signal to organise spindle assembly around chromosomes. However, the local action of Ran is determined by its interaction with intracellular structures such as the NPC in interphase, centrosomes and the spindle during mitotic spindle formation, and chromatin at telophase. The relative importance of the gradient in soluble Ran-GTP concentration in providing a spatial signal as opposed to the action of Ran at pre-defined sites is not yet certain and may differ between cell types. It may also change during progression through mitosis, for instance when the function of Ran switches from microtubule organisation at a distance from chromatin in prometaphase to nuclear envelope formation on chromatin at telophase.

## Conclusion

We have analysed the dynamic localisation of Ran fused to a fluorescent protein in live human cells. Using mutants of Ran stabilised in either the GTP-bound or nucleotide-free state, we show that Ran interacts dynamically with specific subcellular structures depending on its nucleotide-bound state. We infer that Ran-GTP generated at chromatin is highly mobile and interacts with distal structures that are involved in nuclear transport and mitotic spindle assembly. These results support a model for the spatial action of Ran in which Ran-GTP provides a low-resolution signal of the presence of chromosomes while its localised sites of action are specified by the subcellular structures with which it interacts.

## Methods

### Expression constructs

Ran^WT^, Ran^T24N^, Ran^Q69L ^and GST constructs in the pEGFP-1 expression vector (Clontech) were as previously described [15]. These vectors expressed GFP as an N-terminal fusion with the proteins. The pU6YH-RFP-H2B plasmid, generated by subcloning the histone H2B sequence downstream of DsRed (RFP) in the pU6YH vector, was a gift from Dr. I.M. Porter and Dr. M. Posch (University of Dundee).

### Cell culture, transfection and imaging

Human cells were cultured on coverslips, transfected with plasmids, and assembled into a live-cell chamber as previously described [26]. The chamber was connected to a thermal control unit to maintain a temperature of 37°C, then mounted onto the stage of a DeltaVision Spectris microscopy workstation controlled by softWoRx software (Applied Precision), consisting of an Olympus IX70 inverted fluorescence microscope equipped with a computer-controlled stage, a Photometrics CoolSNAP HQ digital camera and a 10 mW 488 nm solid-state laser. Fluorescence images were acquired with a 60× objective. At least 50 cells were observed for each experiment, except for mitotic cells expressing GFP-Ran^Q69L^, where at least 10 cells were observed for each experiment.

### Fluorescence photobleaching

For fluorescence recovery after photobleaching (FRAP) experiments, three images of the cell were captured prior to bleaching, then the laser was focussed to a diffraction-limited spot and spot bleaching performed with a single 20 ms stationary pulse. Sixty post-bleach images (50 ms exposure) were captured during the subsequent recovery phase. For faster-recovering cells, a 'fast-as-possible' protocol was employed, in which the camera shutter remained open and fluorescence images were sampled every 50 ms. For slower-recovering cells, the four-phase sampling protocol was employed, as previously described [26]. Analysis of FRAP data was performed using softWoRx software. Fluorescence intensity values within the bleach spot were determined for each of the time values and expressed as a fraction of the mean of the three pre-bleach values. Fractional intensity values during the recovery phase were fit to a 2D-diffusion algorithm [30], yielding recovery half-time (t_1/2_) and mobile fraction (MF) values. At least 10 cells were analysed for each condition and t_1/2 _values are expressed ± 1 standard deviation.

For fluorescence loss in photobleaching (FLIP) experiments, three pre-bleach images of each cell were taken, then one hundred cycles of laser-bleaching (20 ms) and imaging were performed. Image data were exported from softWoRx as MPEG files, from which frames were imported into Improvision Openlab software as separate layers for analysis.

To quantitate the loss of fluorescence, signal intensities within the defined region of interest (nucleus or chromatin) at different time points were determined, and expressed as percentages relative to the first pre-bleach image. The ratio module of Openlab was used to divide selected grayscale images from the FLIP timecourse by a pre-bleach image, generating a set of grayscale ratio images. These were then re-coloured to make a series of ratio images that correspond to a spectrum.

## Authors' contributions

JRAH generated and analysed the data on localization, FRAP and FLIP. WJM made GFP-fusion constructs, and generated and analysed FLIP data. PRC was the principal investigator and was responsible for supervision of the study and writing the manuscript together with JRAH. All authors read and approved the final manuscript.

## Supplementary Material

Additional file 1 **FLIP analysis of GFP-Ran^Q69L^**. Images of a cell expressing nuclear GFP-Ran^Q69L ^and after successive cycles of photobleaching within a defined spot within the nucleus. The cell was subjected to 100 cycles of laser-bleaching, each of 20 ms duration. Image data were exported from softWoRx as MPEG files, from which frames were imported into iMovie and Quicktime software. Click here for file
